# Brain responses to food cue viewing in pregnant women with and without gestational diabetes mellitus

**DOI:** 10.1038/s43856-026-01792-3

**Published:** 2026-08-06

**Authors:** Mariana Treviño Montemayor, Anna Lesniara-Stachon, Dan Yedu Quansah, Sybille Schenk, Martin Heni, Stephanie Kullmann, Ulrike Toepel, Micah M. Murray, Chrysa Retsa, Jardena J. Puder

**Affiliations:** 1 Obstetric Service, Department Woman-Mother-Child, Lausanne University Hospital and University of Lausanne, Lausanne, Switzerland; 2 Service of Endocrinology, Diabetes and Metabolism, Lausanne University Hospital and University of Lausanne, Lausanne, Switzerland; 3 Department of Internal Medicine I, Division of Endocrinology and Diabetology, University of Ulm, Ulm, Germany; 4 Department for Diagnostic Laboratory Medicine, Institute for Clinical Chemistry and Pathobiochemistry, Eberhard Karls University Tübingen, Tübingen, Germany; 5 Institute for Diabetes Research and Metabolic Diseases (IDM) of Helmholtz Munich at the University of Tübingen, Tübingen, Germany; 6 Internal Medicine IV, Department of Diabetology, Endocrinology and Nephrology, University Hospital Tübingen, Tübingen, Germany; 7 German Center for Diabetes Research (DZD), Tübingen, Germany; 8 The Radiology Department, Lausanne University Hospital and University of Lausanne, Lausanne, Switzerland; 9 The Sense Innovation and Research Center, Lausanne and Sion, Switzerland

**Keywords:** Cognitive neuroscience, Gestational diabetes

## Abstract

**Background:**

Pregnancy-induced food cravings are associated with excessive gestational weight gain and gestational diabetes mellitus (GDM). This study aimed to measure brain responses to food pictures with varying fat and carbohydrate content during pregnancy in women with and without GDM.

**Methods:**

To characterize brain-metabolism coupling, we recorded visual evoked potentials (VEPs) in pregnant individuals with and without GDM (24-36 weeks gestation) while they viewed food pictures varying in fat and carbohydrate content. Eighteen women with untreated GDM and 21 healthy pregnant controls participated. HbA1c levels were obtained from blood samples. Statistical analyses assessed group differences and interactions.

**Results:**

Within the initial ~150 ms post-stimulus onset, VEPs exhibited Group × Fat (*p* = 0.047, ηp^2^ = 0.103), but no Group × Carb, interactions: Healthy pregnant controls showed stronger responses to images of high- versus low-fat foods, whereas individuals with GDM did not. Source estimations localized these effects to brain regions associated with control, attention, and reward. Moreover, brain responses correlated with HbA1c levels in GDM (*p* = 0.01, ηp^2^ = 0.351), but not with pre-pregnancy BMI in either group.

**Conclusion:**

These results provide evidence of links between spatiotemporal brain responses to visual food cues and metabolic health in pregnancy.

## Plain Language Summary

As women often have food cravings in pregnancy, the aim of this study was to investigate how, during pregnancy, the brain of women with adverse metabolic health who have gestational diabetes mellitus (GDM) and thus high blood sugar during pregnancy responds to viewing food pictures compared to healthy pregnant women. A total of 39 pregnant women viewed food pictures with different fat and carbohydrate content. We used non-invasive electroencephalography to record their brain activity. Healthy pregnant women showed stronger brain responses to high-fat compared to low-fat foods, while women with GDM did not make this distinction. Activated brain regions also differed between groups. In women with GDM, brain responses were linked to blood sugar levels. These findings suggest that metabolic health may influence how the pregnant brain reacts to food viewing, which may affect eating behavior and pregnancy outcomes.

## Abstract (web summary)

Treviño Montemayor, Lesniara-Stachon et al. investigate visual food-cue processing in late pregnancy in women with gestational diabetes and healthy controls using electrical neuroimaging. In response to viewing pictures of different fat and carbohydrate content, early brain responses and activated brain regions differ between both groups.

## Introduction

Pregnancy is a period marked by significant hormonal and metabolic changes that influence food cravings and gestational weight gain (GWG)^1–3^. Food cravings, defined as an intense desire to consume specific foods alongside a considerable challenge to resist them^4^, are highly prevalent during pregnancy. They affect up to 80% of women by the end of the second trimester^5^. Hormonal and metabolic changes, including pronounced increases in progesterone and in insulin resistance are thought to contribute, but precise biological and neural mechanisms driving cravings to different food contents in humans remain poorly understood^6^.

In the third trimester of pregnancy, individuals with gestational diabetes (GDM) report twice as many cravings for sweet or sweetened products as those with normal glucose tolerance^5^. Food cravings during pregnancy are associated with increased energy intake and GWG^1–3^. Excessive GWG is linked to adverse perinatal outcomes, including pre-eclampsia, pregnancy-induced hypertension, GDM, cesarean section, preterm birth, and infants born either small or large for gestational age^7^.

GDM is the most common metabolic complication in pregnancy, affecting approximately 16% of pregnant women worldwide^8^ and is linked to substantial perinatal morbidity^9^. GDM is defined as a glucose intolerance first diagnosed during pregnancy, without fulfilling the criteria of overt diabetes^10^. Women diagnosed with GDM are characterized by increased insulin resistance^11^ and face increased long-term metabolic risks, including a 7-10-fold higher risk of developing T2DM^12^, and a 2-fold elevated risk for cardiovascular disease^13^. Understanding how metabolic health influences food-related brain responses during pregnancy is, therefore, critical.

Outside of pregnancy, evidence shows that food consumption is not only regulated by brain circuits involved in the regulation of hunger and satiety, but also circuits involved in reward processing^14^. Alterations in dopaminergic^15,16^ and opioid neurotransmission in brain regions related to reward processing are associated with obesity (OB) and may promote overeating to compensate for decreased hedonic responses^17,18^. Visual exposure is often the first sensory contact with food, and neuroimaging studies have increasingly investigated the neural mechanisms underlying visual food cue responses and their influence on eating behavior and metabolic health.

Functional MRI (fMRI) studies in OB suggest altered brain responses to high-calorie food cues in regions involved in reward processing, including the insula, amygdala, nucleus accumbens, and orbitofrontal cortex (OFC), as well as reduced activity in cognitive control areas, including the anterior cingulate cortex and prefrontal cortex, compared to normal-weight (NW) controls. These responses may be predictive of future weight gain^19–22^. The OFC plays a key role in value-based decision-making, important for integrating reward-based signals^23^. However, findings are mixed, with some studies reporting attenuated responses in reward-related regions in individuals with higher BMI^24,25^.

Electroencephalography (EEG) studies offer a particular advantage by providing insights into the temporal aspects of responses to food cues, allowing for the differentiation between early, more automatic processing and later, more controlled stages of information processing^26^. Biologically relevant food stimuli are discriminated rapidly, within 100–200 ms^27^. Outside of pregnancy, individuals with overweight (OW), compared to those with NW, show weaker early brain responses to food pictures, while later, more conscious, stages of information processing did not differ^28^. Additionally, the strength of early responses to energy-dense high-fat food cues in regions associated with reward valuation and control mechanisms over food intake control have been associated with higher BMI^29^. While many studies report reduced reactivity in cognitive control networks among individuals with OB, both heightened and attenuated responses in reward systems have been described, potentially reflecting individual differences in genotype, satiety state, or the timing of brain responses (in case of EEG)^30–32^.

Beyond weight status, metabolic factors such as glucose and insulin levels also modulate brain responses to food cues^33–35^. For example, insulin levels are particularly related to changes in food cue responses postprandially in the orbitofrontal cortex^35^. fMRI studies show that even mild hyperglycemia ( ~ 7.2 mmol/l), compared to normoglycemia (~5.3 mmol/l), results in decreased activity in the hypothalamus and putamen in individuals with NW, while those with OB exhibited increased activation in reward-related regions, such as the insula, putamen, and prefrontal cortex, without any suppressive effect on hypothalamic activity^33^. These findings suggest that individuals with OB exhibit heightened brain responses to visual food cues in reward-related brain regions during even mild hyperglycemia, without any inhibitory effect on brain regions responsible for the regulation of hunger, satiety, and food intake when compared to individuals with NW.

Similarly, non-pregnant individuals with type 2 diabetes mellitus (T2DM) exhibit increased activation in the insula, OFC, and basal ganglia in response to food pictures compared to healthy controls^34^. Notably, increased activity in the more cortical regions, such as the insula and OFC, correlates with better dietary adherence and self-efficacy, while subcortical activation (e.g., amygdala, caudate, putamen, and nucleus accumbens) is linked with more emotional eating and poorer dietary adherence^34^.

To date, however, information about how the brain processes food during pregnancy is scarce, and data in humans are lacking. To address this gap, we investigate maternal brain responses to visual food cues during pregnancy and their relationship with metabolic health. Using EEG, we compare brain responses to food images varying in fat and carbohydrate content in women with GDM and in healthy pregnant controls in the third trimester. We show that early brain responses to food cues are modulated by fat content in healthy participants but not in women with GDM, indicating differences in early automatic processing of food-related stimuli in GDM. In addition, brain responses to different food categories are associated with HbA1c levels in women with GDM, linking food processing to glycemic control. Differences in activation are observed in regions associated with reward, attention, and cognitive control. Our findings indicate that during pregnancy, metabolic health differences are associated with distinct early brain responses to food cues, which may contribute to food-related behavior during late pregnancy.

## Methods

### Participants, inclusion, and exclusion criteria

Details of the study procedures have been previously published^36^. Seventy-one primi- and multi-parous women in the third trimester of pregnancy participated in the study. We studied two groups: Women with GDM were diagnosed between 24 and 32 gestational weeks, according to the International Association of Diabetes Pregnancy Study Group^37^. They were recruited at the GDM clinic in the Lausanne University Hospital. Healthy pregnant controls (HCO) were recruited at the hospital’s ambulatory maternity clinic or through flyers. Inclusion criteria for HCO comprised a pre-pregnancy BMI of ≤25 kg/m^2^, with no history of GDM, no significant dietary or weight changes exceeding 5 kg in the last 5 years (excluding pregnancy), as well as maintaining weight gain within the recommended pregnancy ranges, established by the National Academy of Medicine, previously named Institute of Medicine^38^.

Exclusion criteria for both groups were the following: pre-existing diabetes, diagnosed eating disorders (Anorexia Nervosa, Bulimia Nervosa, and Binge-Eating Disorder), uncontrollable nausea and vomiting, adherence to a vegetarian/vegan diet (for the choice of the food pictures), current insulin treatment, participation in an intervention study, ongoing psychiatric medication treatment, or active suicidal thoughts. Due to poor quality EEG data, especially at the beginning of the study, our analysis was restricted to 18 pregnant women with GDM and 21 HCO (see “EEG recording and pre-processing” for details). All participants provided a signed informed consent. The study protocol was approved by the Human Research Ethics Committee of the Canton de Vaud (CER-VD 2021-01976).

### Data collection and visit

After signing the informed consent and before the visit, participants were contacted by phone to complete data regarding their medical history and lifestyle behaviors, and were then asked to complete validated online questionnaires sent through the Research Electronic Data Capture (REDCap®). Women were asked to eat a balanced meal at 12:00 h (plate based on the Swiss Society for Nutrition recommendations^39^) to minimize the bias of different satiety levels during the visit. The visit during pregnancy (at 24–36 weeks gestational age) took place in the Lausanne University Hospital in the afternoon, between 13:30 and 16:00 h. The EEG was performed two hours after the standardized meal. For details, please see our study protocol^36^.

#### Measures

##### Sociodemographic and medical characteristics

Data on maternal socio-demographic and medical characteristics were collected in the third trimester of pregnancy by phone, before the in-person visit. These data included age, ethnic origin, educational level, information on smoking status during pregnancy (yes, no, stopped since knowledge of pregnancy), alcohol consumption, and drug use during pregnancy, and gestational weight gain up to the study visit.

### Anthropometric data

Pre-pregnancy weight was self-reported and was obtained via the phone interview. Height and weight of participants, to the nearest 0.1 cm and 0.1 kg, respectively, were measured during their visit using regularly calibrated electronic scales (Seca, Model 7017021094, Hamburg, Germany). Pre-pregnancy BMI was calculated as the ratio of pre-pregnancy weight in kilograms to the square of height in meters (kg/m^2^).

### Collection of blood samples—HbA1c level

HbA1c level (%) of each participant was measured from a capillary blood sample using a validated point-of-care device (Afinion^®^ 2) based on a chemical photometric method with boronate conjugation^40^. This measure reflects the mean concentrations of glucose over the last 2–3 months.

### EEG: apparatus and stimuli

The experiment took place in a sound-attenuated booth (WhisperRoom model 102126E) at 14:00 after a balanced meal based on the recommendations of the Swiss Society of Nutrition at 12:00. Selected visual cues^41^ were presented on a 27-in. LCD monitor (HP EliteDisplay E273q, 2560 × 1440 pixels, 60 Hz).

Participants were presented with four blocks of trials, each comprising 60 pictures: 50 food pictures and 10 non-food objects (e.g., objects of daily use), for a total of 240 pictures (200 pictures of foods and 40 pictures of non-food items). The food and non-food pictures used in this study were selected from a larger food picture database and have previously been rated as highly familiar pictures^41^. Pictures were identical in size and controlled for low-level visual features, such as luminance^27,42^. Each picture was presented for 500 ms, and the order of the blocks was randomized across participants. A central fixation cross was displayed during interstimulus intervals to minimize eye movements.

Food pictures were categorized into four groups based on their fat and carbohydrate content: high-fat/high-carbohydrate (HFHC), high-fat/low-carbohydrate (HFLC), low-fat/high-carbohydrate (LFHC), and low-fat/low-carbohydrate (LFLC). High-fat was defined as a fat content of >5 g/100 g (21.6 ± 13.77 g/100 g) and low-fat of <5 g/100 g (1.1 ± 1.29 g/100 g). High-carbohydrate was defined as carbohydrate content of >15 g/100 g (48.57 ± 21.13 g/100 g) and low-carbohydrate as <15 g/100 g (4.59 ± 4.59 g/100 g). Visual food cues were selected to ensure maximal distinction between food categories.

The entire experimental session lasted approximately 13 minutes. Continuous EEG was recorded using a 128-channel EEG system on the head surface. For this study, visual evoked potentials (VEPs), topographic analyses, and source estimation analyses were performed using only the brain response data evoked by food pictures (see “Data analysis” for details).

### EEG: behavioral task

During the food cue viewing, participants performed a speeded 2-Alternative Forced-Choice (2AFC) discrimination task. Following each image presentation, participants had to indicate whether the presented picture was a food or a non-food object by pressing a button on a response box using their dominant hand (button “1” for food pictures, button “2” for non-food object pictures). To determine the handedness, participants completed the Edinburgh Handedness Inventory^43^. Based on this assessment, 35 participants were right-handed, and 4 were left-handed.

Before the experiment started, the task was explained in detail by the experimenter. Participants also completed a short training session to become familiar with the task. Additionally, brief on-screen instructions were provided before the start of each of the four blocks. Participants were encouraged to take short breaks between blocks in order to minimize fatigue.

Behavioral outcomes included mean response time (milliseconds) for correctly discriminated food and non-food pictures, as well as for each food category. Accuracy (%) was calculated as the percentage of correct trials.

#### Statistics and reproducibility

Sociodemographic and metabolic data analysis were conducted using SPSS (version 29.0.1.0). Descriptive variables were described as means (±SD) or percentages (%) where appropriate (Table 1, supplementary Table 1). To ensure that data quality-related exclusions did not systematically influence the sample composition, we compared women included in the analyses with those excluded (Supplementary Table 1) using unpaired *t*-tests. Continuous variables were normally distributed in each group, as indicated by the Shapiro–Wilk test (*p* ≥ 0.05). Unpaired *t*-tests and Chi-square tests were used to examine group differences in sociodemographic and metabolic data.

**Table 1:** Socio-demographic and medical characteristics of participants

Variable	All women (*N* = 39)	Women with GDM (*N* = 18)	HCO (*N* = 21)	*p*-value
Age (year)	31.8 ± 3.9	31.1 ± 4	32.2 ± 3.9	0.37
Pre-pregnancy weight (kg)	66.5 ± 14.2	74.7 ± 16.5	59.4 ± 6.3	<0.001
Pre-pregnancy BMI (kg/m^2^)	24.4 ± 5.4	27.7 ± 6.2	21.5 ± 2.1	<0.001
Gestational age during visit (weeks)	29.5 ± 3.1	30.1 ± 1.8	28.3 ± 3.4	0.01
Current weight (kg)	76.5 ± 13.6	83.8 ± 15.4	70.2 ± 7.9	0.001
Gestational weight gain up to visit (kg)	10 ± 5.1	9.1 ± 6.5	10.9 ± 3.6	0.29
HbA1c (%)	5.06 ± 0.35	5.18 ± 0.42	4.97 ± 0.25	0.07
**Educational level**				0.02
Incomplete compulsory school	0 (0%)	0 (0%)	0 (0%)	
Compulsory school achieved	0 (0%)	0 (0%)	0 (0%)	
High school	2 (5%)	2 (11%)	0 (0%)	
General and vocational education	15 (39%)	10 (56%)	5 (24%)	
University	22 (56%)	6 (33%)	16 (76%)	
**Ethnic origin**				0.12
Switzerland	17 (43.6%)	6 (33%)	11 (52%)	
Europe West	13 (33.3%)	4 (22%)	9 (43%)	
Europe East	3 (7.7%)	3 (17%)	0 (0%)	
Africa	2 (5.1%)	2 (11%)	0 (0%)	
North of America	2 (5.1%)	1 (6%)	1 (5%)	
Asia	1 (2.6%)	1 (6%)	0 (0%)	
Latin America	1 (2.6%)	1 (6%)	0 (0%)	
**Tobacco during pregnancy**				0.51
No	33 (87%)	15 (83%)	19 (90%)	
Yes	5 (13%)	3 (17%)	2 (10%)	
**Drugs during pregnancy**				NA
No	39 (100 %)	18 (100%)	21 (100%)	
Yes	0 (0 %)	0 (0 %)	0 (0 %)	
**Alcohol during pregnancy**				0.91
No	34 (89%)	16 (89%)	18 (90%)	
Yes, occasional consumption	4 (11%)	2 (11%)	2 (10%)	

Values are presented as mean ± standard deviation or number (%). *P*-values indicate group differences assessed using either a *t*-test or chi-squared test, as appropriate. *NA* not applicable.

### Behavior results analyses

We compared the response time and accuracy to food and non-food pictures between women with GDM and HCO. As the residuals from the mixed ANOVA violated the assumptions of normality (Shapiro–Wilk test, *p* < 0.05), non-parametric tests were used. Specifically, Mann–Whitney *U* tests were used for between-group comparisons, and Friedman tests for within-group comparisons. Kendall’s coefficient of concordance (*W*) was calculated to estimate the effect size (Supplementary Table 3).

### EEG recording and pre-processing

Continuous EEG was acquired using a 128-channel (ANT EEGO my lab system) at 1000 Hz. EEG data pre-processing was performed with the Cartool freeware^44^. EEG signals were filtered (0.1 Hz high-pass, 40 Hz low-pass, and 50 Hz notch), using a fourth-order non-casual Butterworth filter (−24 dB/octave roll-off) in both directions.

Peri-stimulus epochs, spanning −100 to 500 ms relative to picture onset, were averaged for each participant and each food picture category (HFHC, HFLC, LFHC, LFLC) to calculate VEP. Epochs were rejected if the signal in any EEG channel exceeded ±100μV (automated artifact rejection criteria), as well as based on visual controls for eye movement and blinks or other causes of transient noise. Before averaging, deficient recording channels were identified and excluded from further analyses. Using 3-D splines, data from electrodes with artifacts were interpolated in each individual participant before group averaging^45^. Also, data were baseline-corrected using the 100 ms pre-stimulus period and recalculated against the average reference. After pre-processing, 31 participants had to be excluded from further analyses since less than 60% of trials to at least one food category could be retained. After exclusion, the percentage of accepted trials was 88 ± 12 % in women with GDM and 89 ± 11 % for HCO. There were no significant differences between groups or categories (all, *p* > 0.51).

### VEPs analyses

Differences in brain responses to food pictures between GDM and HCO groups were assessed using an electrical neuroimaging framework in a multi-step analysis procedure including both local and global electric field measures on the scalp, as previously described^46,47^.

### Global field power analyses

To quantify differences in the amplitude of VEPs between groups and food categories, global field power (GFP) was calculated for each participant and food category^48^. GFP corresponds to the spatial standard deviation of the electric field power across all electrodes and is used to quantify the amount of brain activity at each time point in the field considering the data from all recording electrodes simultaneously^49^.

**Fig. 1:**
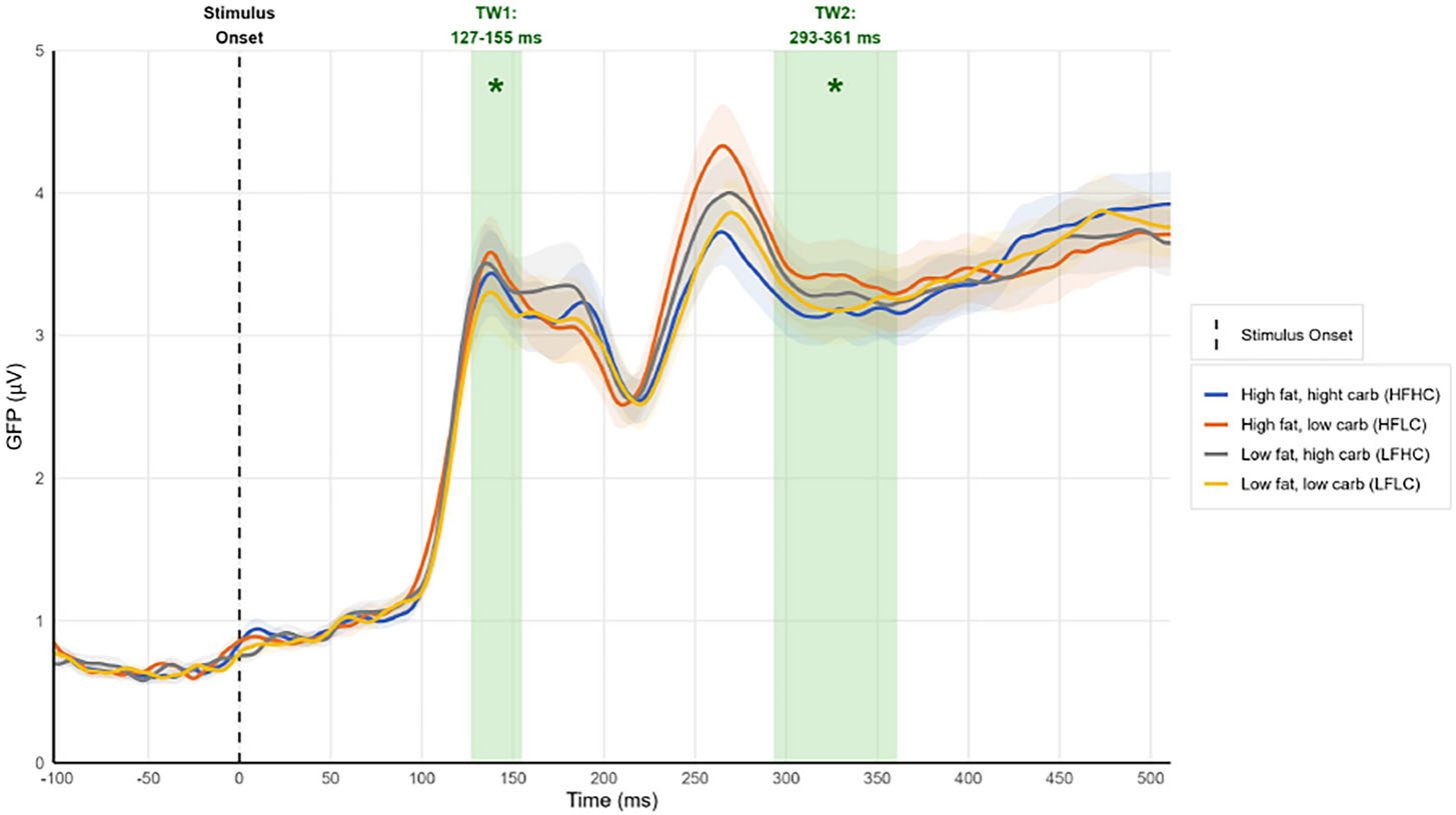
Averaged global field power (GFP) in response to food picture categories. Averaged Global field power (GFP) waveforms for all participants (*n* = 39) and standard error bands are shown separately for each food category across the peri-stimulus interval (−100 to 500 ms). Green bars indicate two time windows (TW1: 127–155 ms; TW2: 293–361 ms), which showed a significant fat x carb interaction (*p* < 0.05) in brain responses between fat and carbohydrate content of food pictures. GFP denotes global field power.

VEP brain responses to visual food cues have, to the best of our knowledge, never been studied in pregnancy. Therefore, with an exploratory perspective, we first compared the GFP as a function of time using a two-way repeated measures analysis of variance (rmANOVA) with fat (high-fat vs. low-fat) and carbohydrate (high-carb vs. low-carb) as the within-subject factors (Fig. 1). This analysis, performed using SPSS (version 29.0.1.0), identified two time windows with significant fat × carb interaction: Time Window 1 (TW1), 127–155 ms post-stimulus and Time Window 2 (TW2), 293–361 ms post-stimulus. These time windows were used for all subsequent analyses.

**Fig. 2:**
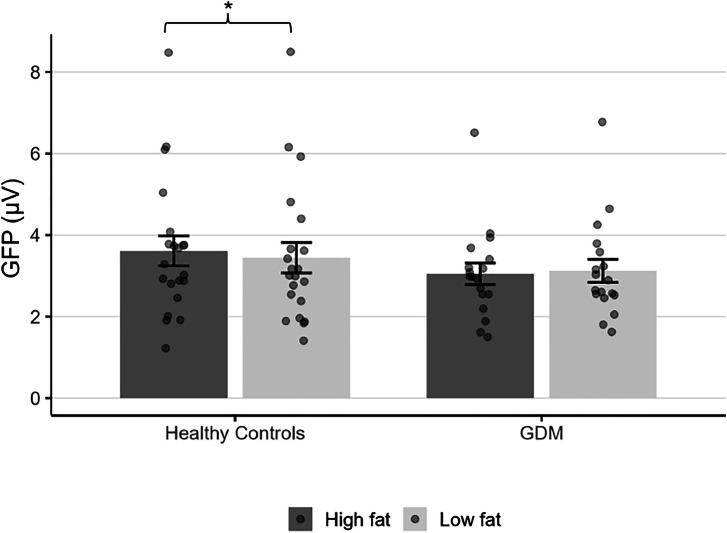
Global field power (GFP) to food pictures varying in fat content over the time window of 127–155 ms after stimulus onset in women with GDM and healthy controls. Data are presented as mean ± SE, with dots indicating individual participant data; *n* = 39 participants (Healthy controls *n* = 21, GDM *n* = 18). Statistical analysis was performed using a mixed-model rmANOVA followed by Bonferroni-corrected pairwise comparisons (two-sided). A significant fat × group interaction was observed (*p* = 0.047). Post hoc comparisons showed that only healthy controls exhibited stronger brain responses to high-fat food pictures compared to low-fat food pictures (*p* = 0.04).

In a second step, we investigated group differences in brain responses by calculating the average GFP values within each time window for each participant and food category. For each time window, we performed a two-way mixed-model ANOVA (mixed ANOVA) with fat (high-fat vs. low-fat) and carbohydrate (high-carb vs. low-carb) as the within-subject factors and group (GDM vs. HCO) as the between-subject factor. These analyses were also performed using SPSS (version 29.0.1.0). Where significant interactions were observed, we conducted post hoc comparisons with Bonferroni correction to assess within- and between-group differences (Fig. 2).

In an additional step, we investigated the associations between metabolic health (HbA1c levels and pre-pregnancy BMI) and brain responses. For each time window, we conducted an analysis of covariance (ANCOVA) to measure correlations between the HbA1c, the groups, and the food picture conditions. In case the ANCOVA indicated a significant interaction with the covariate, we performed Pearson correlation analyses between mean GFP values for each food category and the covariate. To assess whether the association of HbA1c levels and brain processing of different food cues was independent of sociodemographic and medical factors differing between groups (see Table 1).

### Topographical analyses

In a third step, we investigated topographical differences in the electric field at the scalp surface between participant groups using a topographic clustering analysis and a hierarchical clustering algorithm implemented in Cartool^50^ on the post-stimulus group-averaged VEPs to the four food categories and the two groups. This analysis identifies periods of stable scalp electric field topographies (“template maps”) and is insensitive to variations in response strength across the four food categories, as the data are first normalized by their instantaneous GFP. The optimal number of clusters was determined using the meta-criterion^51^. Once the pattern of maps was defined at the group-average level, a fitting procedure was used where each time point of each participant’s event-related potential is assigned the template map that presented the strongest spatial correlation^50^. This fitting provides a quantification of the presence of each map over a specific time window (ms), which was analyzed using a mixed ANOVA with factors of food categories, group, and maps. These results indicated whether the VEPs to a particular food category were predominantly described by one or more particular template maps, in extension indicating differences in the intracranial generator configurations of these VEPs^46^.

### Source estimation analyses

In a final step, we then explored the intracranial sources of these VEPs in response to the four different food categories. Source estimations were performed using a distributed linear inverse solution using the local autoregressive average regularization approach (LAURA)^52–54^ implemented in Cartool. The head model and lead-field matrix were generated based on the Montreal Neurological Institute’s average brain (available from https://github.com/DenisBrunet/Cartool) using the Spherical Model with Anatomical Constraints^55^. LAURA provides current density measures as output, with scalar values at each of 3058 nodes in the solution space. For statistical analyses, VEPs were averaged across time within each of the two previously defined time windows (TW1: 127–155 ms and TW2: 293–361 ms post-stimulus) for each participant and viewing condition (food categories). Source activity (in μA/mm^3^) was then computed for each node in the solution space in every participant. To compare activity between groups and viewing conditions, we performed mixed ANOVAs using STEN toolbox version 2.0, developed by Jean-François Knebel and Michael Notter (10.5281/zenodo.1164038). Differences in source activity were considered significant when (a) activity differed as a function of group and/or viewing condition at the level of individual nodes (*p* < 0.05) and (b) when at least ten contiguous nodes surviving the statistical threshold formed a reliable cluster^56,57^. This spatial extent criterion is based on simulations of random contiguous cluster sizes using an approach previously described to control for false positive rates at the individual node level^58^. To further investigate differences in activity between groups and viewing conditions within these clusters, we performed a mixed ANOVA with fat (high-fat vs. low-fat) and carbohydrate (high-carb vs. low-carb) as the within-subject factors and group (GDM vs. HCO) as the between-subject factor using SPSS. Where significant interactions were observed, we conducted post hoc comparisons with Bonferroni correction to assess within- and between-group differences (Fig. 4 and Supplementary Table 3).

For the primary analyses in this study, we focused on the earliest differential response (TW1), corresponding to early perceptual and reward-related processing. Prior research shows that biologically relevant food stimuli are discriminated within 100–200 ms^27^. These early processes are thought to reflect automatic evaluations related to reward valuation and behavioral relevance, supporting their importance in the context of food perception. Focusing on this early window may allow us to better understand the initial, more automatic differences in food cue processing associated with metabolic health during pregnancy.

## Results

### Characteristics of study participants

The mean age, pre-pregnancy weight, and pre-pregnancy BMI of the analyzed participants (*N* = 39) were 31.7 ± 3.9 years, 66.5 ± 14.3 kg, and 24.4 ± 5.4 kg/m, respectively. Most of the participants in the GDM group were overweight (*n* = 5, 28%) or obese (*n* = 7, 39%). The majority of women were non-smokers (86.84%) and abstained from alcohol during pregnancy (87.18%) (Table 1). In terms of metabolic health, women with GDM had a higher pre-pregnancy weight and BMI, as well as current weight (all *p* ≤ 0.001) compared to HCO, and a tendency for higher HbA1c levels (*p* = 0.065).

### Behavioral results

Regarding the behavioral responses to the 2AFC discrimination task, both groups reacted faster to food pictures compared to non-food object pictures (GDM: 496.3 vs. 567.3 ms; HCO: 512.7 vs. 592.6 ms; all, *p* < 0.001) and had similar response accuracy (GDM: 97.3% vs. HCO: 98.1%, *p* = 0.23). When comparing between-group (GDM vs. HCO) reaction times to food (pooled food categories) and non-food object pictures, no significant differences were observed (all *p* ≥ 0.13). Within-group differences in response to the food categories were observed only in the HCO group, *x*^2^(3) = 18.6, *p* < 0.001, with a moderate effect size (Kendall’s *W* = 0.3). Post hoc comparisons with Bonferroni correction (*α* = 0.008) indicated that response times for LFLC were faster than for the HFLC and LFHC categories in the HCO (all, *p* ≤ 0.005). (Supplementary Table 2).

### Global field power results

We ran a mixed-model rmANOVA with the averaged GFP values within each time window for each participant and food condition to investigate group differences in the strength of brain responses. The results for TW1 (127–155 ms) showed no significant main effects of fat, carb, or group (all, *p* > 0.4), but an interaction of fat × carb (*F*(1,37) = 7.591, *p* = 0.009, *η*_p_^2^ = 0.17) was observed (see Fig. 1). Post hoc comparisons, adjusted for multiple comparisons (Bonferroni), showed overall stronger responses to HFLC and LFHC food categories compared to LFLC category (HFLC: 3.398 ± 0.222 μV; LFHC: 3.378 ± 0.237 μV vs. LFLC: 3.190 ± 0.250 μV; all, *p* < 0.026). Additionally, a fat × group interaction was observed (*F*(1,37) = 4.237, *p* = 0.047, *η*_p_^2^ = 0.103) (see Fig. 2). The post hoc comparisons showed an effect of fat only in the HCO group (*F*(1, 20) = 4.557, *p* = 0.039, *η*_p_^2^ = 0.11): Specifically, women in the HCO group had stronger responses to high-fat food compared to low-fat food images (HF: 3.61 ± 0.317 μV vs. LF: 3.446 ± 0.328 μV; *p* = 0.039). This effect of fat was not observed in women with GDM (HF: 3.052 ± 0.343 μV vs. LF: 3.123 ± 0.354 μV; *p* = 0.413).

The result of the mixed-model rmANOVA on the averaged GFP values across TW2 (293–361) showed no significant main effects of fat or carb (all, *p* > 0.19), but there was an interaction of fat x carb (F(1,37) = 4.682, *p* = 0.037, η_p_^2^ = 0.112). *Post-hoc* comparisons, adjusted for multiple comparisons (Bonferroni), showed overall stronger responses to the HFLC food category compared to the HFHC category across both groups (3.399 ± 0.252 μV vs. 3.155 ± 0.200 μV; *p* = 0.017). No group interactions were observed.

### Associations between global field power and metabolic health

**Fig. 3:**
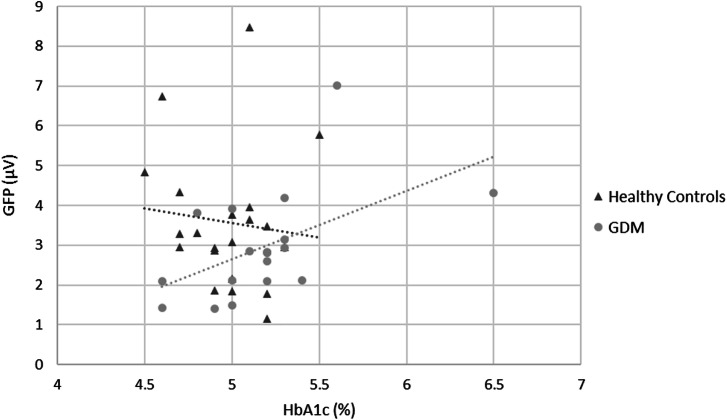
Correlation between HbA1c levels and brain responses (global field power) to high-fat/high-carbohydrate food pictures*.* Correlation between brain responses and high-fat/high-carbohydrate food pictures in TW1 (127–155 ms) was observed in women with GDM, but not in HCO (high-fat/high-carbohydrate: GDM *r* = 0.53, *p* = 0.02; HCO: *r* = -0.11, *p* = 0.65). GFP denotes global field power; GDM denotes gestational diabetes; HCO denotes healthy controls. Results remained significant for GDM after the exclusion of the participant with a high HbA1c value (6.5%) in the GDM group.

Additionally, we conducted an ANCOVA to measure correlations between the groups, the averaged GFP to the food picture conditions, and metabolic health. When investigating the association of the averaged GFP responses for each food category with HbA1c within TW1 (127–155 ms), we observed a significant interaction of fat × carb × group × HbA1c interaction (*F*(1, 37) = 4.152, *p* = 0.049, *η*_p_^2^ = 0.106). This interaction remained significant after adjustment for pre-pregnancy BMI, educational level, and gestational age (all, *p* ≤ 0.048). The post hoc comparisons indicated an interaction of fat × carb × =HbA1c only in women with GDM (*F*(1, 17) = 8.664, *p* = 0.01, *η*_p_^2^ = 0.351), but not in HCO (*p* = 0.750). In women with GDM, but not HCO, a positive correlation between the strength of brain responses and the HbA1c levels was found. This was observed for the majority of food categories (HFHC, HFLC, and LFLC, all *p* ≤ 0.047, trend for the LFHC, *p* = 0.066), Fig. 3). Results remained significant after the removal of the participant with increased HbA1c value (6.5%) in the GDM group. In contrast, no significant interactions were observed between the groups, averaged GFP for the different food categories, and pre-pregnancy BMI.

### Topographical differences

No interaction involving groups was observed in any TW. Thus, these results will not be presented in more detail.

### Source estimations results

**Fig. 4:**
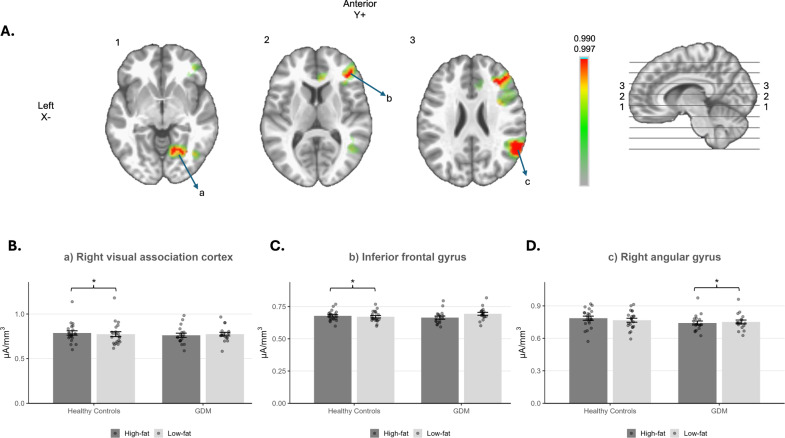
LAURA source estimations over the TW1 (127-155 ms) regarding the viewing of food pictures of different fat content. **A** Axial brain slices illustrating LAURA source estimations over TW1, highlighting significant differences in response to fat between Healthy Control (HCO, *n* = 21) and gestational diabetes mellitus (GDM, *n* = 18) groups. **B** Mean source activation (μA/mm^3^) in the right visual association cortex; HCO participants showed stronger activations for high- versus low-fat conditions (*p* = 0.040, one-sided). **C** Mean source activation in the inferior frontal gyrus; GDM participants exhibited reduced activation to high- versus low-fat stimuli (*p* = 0.001). **D** Mean source activation in the right angular gyrus; HCO participants showed stronger activations for high- versus low-fat conditions (*p* = 0.013). Data in (**B**–**D**.) are presented as mean ± SE, with dots indicating individual participant data. Statistical analyses were performed using Bonferroni-corrected post hoc comparisons following a mixed-model rmANOVA (two-sided unless specified).

**Fig. 5:**
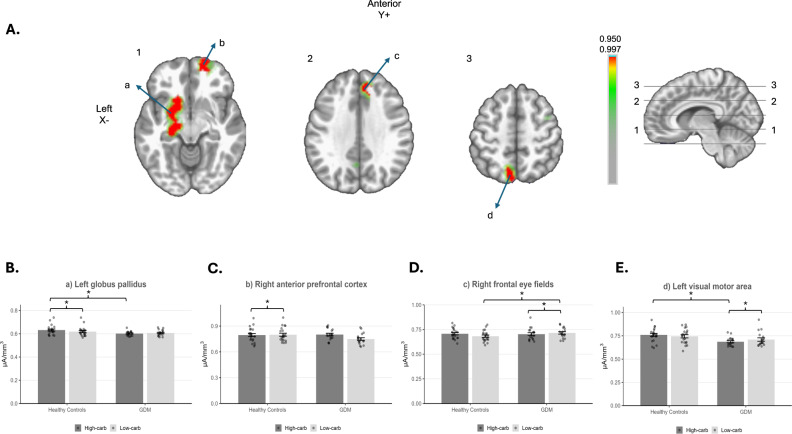
LAURA source estimations over the TW1 (127–155 ms) regarding the viewing of food pictures of different carbohydrate content. **A** Axial brain slices illustrating LAURA source estimations over TW1, highlighting significant differences in response to carbohydrates between Healthy Control (HCO, *n* = 21) and gestational diabetes mellitus (GDM, *n* = 18) groups. **B** Mean source activation (μA/mm^3^) in the left globus pallidus; HCO participants showed higher activation for high- versus low-carbohydrate conditions (*p* = 0.002), and between-group differences indicated greater activation in HCO than GDM in response to high-carbohydrate conditions (*p* = 0.007). **C** Mean source activation in the right anterior prefrontal cortex; GDM participants exhibited higher activation for high- versus low-carbohydrate conditions (*p* = 0.002), and between-group differences indicated greater activation in HCO than GDM under low-carbohydrate conditions (*p* = 0.04). **D** Mean source activation in the right frontal eye fields; HCO participants showed higher activation for high- versus low-carbohydrate conditions (*p* = 0.022). **E** Mean source activation in (**d**) left visual–motor area; GDM participants exhibited lower activation for high- versus low-carbohydrate conditions (*p* = 0.022), and between-group differences indicated greater activation in HCO than GDM under high-carbohydrate conditions (*p* < 0.001). Data in **B**–**E** are presented as mean ± SE, with dots indicating individual participant data. Statistical analyses were performed using Bonferroni-corrected post hoc comparisons following a mixed-model rmANOVA (two-sided).

LAURA-distributed source estimations were calculated separately over TW1 (127-155 ms) and TW2 (293–361). Where significant interactions were observed, post hoc comparisons were conducted with Bonferroni correction. For TW1, significant group differences were observed for the estimated source activity in response to fat, carb, and the interaction of fat × carb (Fig. 4, Fig. 5, and Supplementary Table 2). Regarding differences in response to fat content (Fig. 4), group differences were found within the right visual association cortex (MNI: 44, −68, −12 mm), right angular gyrus (MNI: 52, −60, 20 mm), as well as the right inferior frontal gyrus (pars opercularis) (MNI: 44, 12, 28 mm). Specifically, the HCO group had a stronger activation in the right visual association cortex and right angular gyrus when viewing high-fat compared to low-fat food pictures; differences were not observed in women with GDM (all, *p* < 0.04). In contrast, only women with GDM showed stronger activation of the right inferior frontal gyrus when viewing low-fat food images compared to high-fat food pictures (*p* = 0.001).

Regarding differences in responses to carbohydrate content (Fig. 5), group differences were observed in the left globus pallidus (MNI: −20, 4, −4), right frontal eye field (MNI: 4, 36, 36), left visual motor area (MNI: −4, −76, 52), and the right anterior prefrontal cortex (MNI: 20, 68, −4). Only the HCO group showed stronger activation in the left globus pallidus and the right frontal eye field for high- compared to low-carbohydrate food pictures (all, *p* < 0.02). In the left visual motor area, women with GDM showed stronger activation for low- compared to high-carb food pictures, and in the right anterior prefrontal cortex for high- compared to low-carb food pictures (all, *p* < 0.02); no such effects were observed in the HCO group. Regarding between-group differences, women in the HCO group presented stronger activation in the left globus pallidus and in the left visual motor area for high-carb food pictures, and in the right anterior prefrontal cortex for low-carb food pictures compared to women with GDM (all, *p* < 0.04).

Given our emphasis on the earlier TW, we provide only a brief overview of the TW2 source estimation results, and full results can be found in the Supplementary Table 3. Significant between-group differences were observed for the estimated source activity in response to fat, carb, and the interaction of fat x carb in TW2 (Supplementary Table 3). Differential source activity was localized in regions such as the left visual motor area, right dorsolateral prefrontal cortex, and the right ventral posterior cingulate cortex.

## Discussion

Pregnancy represents a unique period with dramatic hormonal and metabolic changes that are not present outside of the perinatal period. Despite its clinical relevance and the high prevalence of pregnancy-related food cravings, this period remains underexplored. We characterize brain responses to visual food cues during pregnancy and their links to metabolic health. Using EEG, we investigated differences in responses to food pictures varying in fat and carbohydrate content in women with GDM and HCO in the third trimester of pregnancy. Analyses of the recorded VEPs, using an electrical neuroimaging framework, allowed us to differentiate between effects due to modulations in response strength from those due to modulations in response topography; the latter of which is a direct indicator of modulations in the underlying configuration of active brain networks. During early, still predominantly automatic, visual processing of food cues (127–155 ms post-stimulus onset), only women in the HCO group showed differential responses according to fat content, with stronger responses to high-fat compared to low-fat foods. Additionally, only women with GDM exhibited a positive correlation between the strength response to different food categories and HbA1c. Although no head-surface differences in response topography were observed between both groups, source reconstructions over this same time period identified differing regional patterns of neural activity in women with GDM vs. HCO, depending on the content of the food cues in regions associated with control, attention, and reward.

Early perceptual and reward-related processing is thought to reflect automatic evaluations related to reward valuation and behavioral relevance, supporting their importance in the context of food perception. During early visual processing, brain responses in women with GDM were not modulated depending on whether high-or low-fat foods had been viewed. In contrast, the HCO showed stronger brain responses to high- compared to low-fat food pictures during this same early window, consistent with a greater salience to high-fat food cues. This finding aligns with previous literature demonstrating that the energetic value of foods, such as fat, is rapidly processed by the brain within the initial 100-200 ms post-stimulus onset^27^. Based on our results, this early sensitivity to food content is reduced in women with GDM and may reflect a disruption in the early perceptual salience or attentional allocation. Similarly, in the behavioral task, when women had to press a button on a response box (food vs. non-food), only the HCO group showed a difference in response time across food categories. The absence of this behavioral effect in the GDM group and the attenuated VEP responses suggest a lower ability to discriminate energetic food components, such as higher fat content, during early cognitive processing. A reduced ability to discriminate food content in visual cues may have implications for eating behavior during early pregnancy by contributing to maladaptive patterns such as overeating and disrupted hunger or satiety cues that lead to subsequent excessive GWG^59,60^, which is associated with adverse outcomes for both mother and child^61^. As most women with GDM were OW (27%) or OB (39%), similar absolute GWG between groups may indicate relatively higher than recommended weight gain in the GDM group preceding this rather late third-trimester visit. Future studies should investigate the processing of food cues earlier in pregnancy to determine whether differences in brain responses can already be observed at earlier stages of pregnancy and if the responses are associated with subsequent GWG trajectories.

We observed an association between HbA1c levels, a surrogate marker for average blood glucose levels over the last 2–3 months, and early brain responses to food cues in pregnancy only in women with GDM. This suggests that, as opposed to HCO, chronically higher glucose levels, even close to normal range, may dysregulate brain responses and be related to stronger brain responses to food pictures. Of note, in individuals with HbA1c levels close to the upper normal range, the large majority of the HbA1c value is contributed by postprandial values. Women with GDM (independent of BMI) have increased peripheral insulin resistance compared to healthy pregnant women^11^. Studies in non-pregnant populations suggest that peripheral insulin resistance is associated with brain insulin resistance, which attenuates insulin’s central effects on food-related neural activity and influences the coupling between peripheral metabolic status and brain responses to food cues^62^. This may differentially impact the relationship between glucose control, insulin resistance, and brain responses to food cues^33^. Consequently, the associations between HbA1c and brain responses observed only in women with GDM may reflect underlying metabolic dysregulation, although this interpretation cannot be confirmed, as insulin resistance was not measured in the present study. In addition, EEG recordings were performed in a standardized postprandial and thus satiated state, which may potentially influence brain responses through acute satiety-related processes and ongoing physiological and glycemic responses to the previous food intake. While no previous studies have investigated this in pregnant populations, evidence from non-pregnant individuals with T2DM shows increased responses to food pictures compared to healthy controls in regions associated with reward processing and salience^34^. These findings may be relevant in the context of metabolic dysregulation and reward-related processing, despite being observed in a different physiological context. Other fMRI studies indicate that acute hyperglycemia is linked to food cue reactivity and may lack a suppressive effect or even display a stimulatory effect on brain activity in individuals at higher metabolic risk: Individuals with normal weight, during mild hyperglycemia (~7.2 mmol/l), as observed in the postprandial state, when compared to the normoglycemic state (~5.3 mmol/l), exhibit decreased brain responses in the hypothalamus and caudate, brain regions involved in the regulation of hunger and satiety, subsequently decreasing food consumption. Conversely, among people with obesity, this suppressive effect of glucose on brain activity in the hypothalamus is not observed^33^. Additionally, among people with obesity, hyperglycemia leads to an increase in brain activity in the insula and putamen, brain regions regulating reward-motivation^33^, suggesting that in hyperglycemia, the reward-urge is not only maintained but even reinforced. In obesity, where brain insulin resistance is present^62^, the lack of an attenuation in the activation of hypothalamus and reward-related brain regions may disrupt the homeostatic balance and potentially reduce inhibitory control, contributing to subsequent overeating behavior^63–65^. A similar mechanism related to brain insulin resistance may occur in the more insulin-resistant subjects with GDM, regardless of their BMI. This may be accentuated in later pregnancy when insulin resistance is generally even more increased. Together, these findings provide a relevant metabolic context for understanding how insulin resistance and glycemic variation may interact with brain processing in response to food cues, while the present findings extend this similar evidence to pregnancy.

Over the early TW, we also observed group differences in the brain networks to food cues according to both fat and carbohydrate content: Women with GDM, but not HCO, showed reduced brain activity in the right inferior frontal gyrus when viewing pictures with high- compared to low-fat. This region is involved in attention and inhibitory control^66^. A reduced activation in this early period toward high-fat food pictures may reflect a weaker engagement of inhibitory control processes, potentially influencing later attention allocation or behavioral responses. In addition, differential responses to fat content were observed in the right angular gyrus and right visual cortex, where HCO showed stronger activation for high- versus low-fat pictures, a pattern not observed in women with GDM. The angular region has previously been implicated in attentional shifting towards cues with high salience, particularly those associated with value or memory^67,68^, suggesting that this region may support early evaluative processing of high palatable food cues. Regarding the response to carbohydrate content, HCO, but not women with GDM, presented a stronger activation for high versus low carbohydrate in the left globus pallidus and right frontal eye fields. The globus pallidus is a subregion of the basal ganglia, a key structure involved in reward-related processes^69^. A previous meta-analysis investigating the role of basal ganglia in food reward processing found that food cues activate the lateral globus pallidus in healthy adults^70^. The absence of this differential response in women with GDM may reflect altered reward processing in response to different carbohydrate food content. Additionally, the HCO group showed greater activation in the right anterior prefrontal cortex when viewing low- compared to high-carbohydrate food pictures, whereas women with GDM showed stronger activation in this region for high- compared to low-carb food pictures. This may reflect a possible compensatory mechanism or altered regulatory strategy in response to carbohydrate cues, possibly involving the integration of cognitive processes such as internal evaluation or strategic control^71^. These results align with findings from other EEG studies in non-pregnant populations. A study found a reduced N1 amplitude to food cues in the 130–190 ms TW after stimulus onset among non-pregnant individuals with overweight, compared to healthy controls^28^. This is thought to reflect reduced visual processing, likely due to reduced early attentional allocation to reward-related cues (pleasant food pictures) among overweight participants. A systematic review of findings from neuroimaging studies also highlights attentional biases to food cues in individuals with obesity^72^.

To the best of our knowledge, this study provides the first evidence on how women process food pictures during pregnancy, a state of increased prevalence of food cravings, and how these processes differ between HCO and women with GDM. While the findings of this study provide valuable insights into potential patterns of brain activity related to adverse metabolic health outcomes, it is important to interpret them cautiously due to certain limitations. One limitation is the relatively small sample size, but still comparable to other EEG studies using similar designs outside of pregnancy. To our knowledge, this is the first exploratory study to investigate brain responses to food cues in late pregnancy. Replication in larger and more diverse cohorts is needed to confirm the robustness and generalizability of these findings. Additionally, HbA1c levels in late pregnancy may be less reliable and may underestimate maternal glycemia, particularly among women with lower hemoglobin levels^73^, potentially influencing the interpretation of our findings. Future studies should therefore consider including additional measures of insulin resistance, such as HOMA-IR, Matsuda, the insulin-sensitivity-index, or measures of glycemic control, such as venous glucose measures or continuous glucose monitoring (CGM), as long as they would not impact the procedure. CGM, for example, could provide more detailed glycemic profiles in future studies; however, it has limited validation in pregnancy at lower glucose levels than those seen in subjects with diabetes and has shown reduced reliability during initial days of sensor use^74^. Postprandial examination of brain responses to food cue viewing, though reducing the potentially confounding hunger-related responses, could be associated with more pronounced differences in glycemia between groups and may have accentuated some of our findings. If prolonged examination in the fasting state, in addition to the postprandial state, could be feasible in this population, this would expand our knowledge in pregnancy. Finally, replication of the inverse solution results using neuroimaging techniques with higher spatial resolution than EEG would strengthen the spatial specificity of these findings, although such approaches may be challenging to implement in pregnant populations.

Late pregnancy is a state of increased insulin resistance and food cravings. In summary, we demonstrate that in late pregnancy, there are distinct temporal dynamics and spatial patterns of brain responses to different visual food cues between women with GDM and HCO. Our findings also suggest that, in women with GDM, glucose control (assessed through HbA1c) interacts with how the brain processes visual food cues in this period of heightened vulnerability. We observed group differences in the early, automatic processing of food cues, reflected in the activation of regions associated with control, attention, and reward, depending on the food content. These insights open possibilities for interventions beyond conventional dietary and lifestyle strategies to limit excessive gestational weight gain and other adverse metabolic health outcomes. Neurocognitive interventions that strengthen attention and inhibitory control may offer innovative ways to modulate neural processing of food cues and ultimately improve maternal and fetal health.

## Supplementary information

Transparent Peer Review file

Supplementary Information File

Description of Additional Supplementary files

Supplementary Data 1

## Data Availability

The source data for Figs. 1–5 can be found in the Supplementary Data file. The datasets generated and analyzed during the current study are available from the corresponding author (J.J.P) upon reasonable request. Access will be granted following review of a request, which should include a brief description of the proposed research use, confirmation of relevant ethical approval where applicable, and agreement to data use conditions. These conditions prohibit data redistribution and restrict use to the approved scope request. All requests will be reviewed on a case-by-case basis. A response will typically be provided within 2 weeks of receipt of a complete request.
