# Supplementary material for: Brain responses to food cue viewing in pregnant women with and without gestational diabetes mellitus
Source: Commun Med (Lond). 2026 Aug 6;6:426. doi: 10.1038/s43856-026-01792-3 (PMC13448487; doi:10.1038/s43856-026-01792-3)
Supplement: Supplementary file 1 [file 43856_2026_1792_MOESM1_ESM.pdf]

nature portfolio

Peer Review File

Brain responses to food cue viewing in pregnant women with and without gestational diabetes mellitus

Corresponding Author: Professor Jardena J. Puder

This file contains all reviewer reports in order by version, followed by all author rebuttals in order by version.

Version 0:

Reviewer comments:

Reviewer #1

(Remarks to the Author)
This study aimed to investigate the impact of gestational diabetes mellitus (GDM) on maternal brain responses to visual food cues in pregnancy. The association of hbA1c/BMI and VEPs was examined. The authors try to fill in the gap of understanding these conditions effect on the brain responses to visual food cues during pregnancy. The manuscript is easy to follow and well structured.
Major comments:
1. According to Table 1, both groups were well maintained for the hbA1c level, thus yields no significant difference. In the glucose lowering treatment, about 40% were not treated but still well maintained or were they diagnosed using other parameters? So hypothetically, how much effect of GDM is expected to detect with the well maintained hba1c level?
2. In figure 4, it is clearer that only 1 participant is in the abnormal range of the hba1c. The entire association of the GDM group is suspected to be driven by that subject (6.5%) and the one about at the upper edge of normal range (slightly higher than 5.5%). With very few subjects have higher value of hba1c, this association may be quite weak. Together with both groups included in the hba1c association analysis, is it still significant?
3. Similar problem may be applied to obesity status. The authors also introduced many evidences supporting the impact of obesity in neural processing of food cues, but the participants included were merely overweight, please indicate how many were obese. Or the authors may try to give examples and explain that the subtle overweight can also have an impact. However, such effect was not detected in the current study, and this need to be explored.
4. Were any covariates controlled in the statistical analyses? Given the evidences of impact of obesity in neural processing of food cues as the authors listed, and the participants have BMI information, was it considered as a confounding factor? Additionally, in table 1, the educational level was significant different between the two groups, was it controlled?
Minor comments:
1. Please add the effect size of the significant correlations reported in the abstract.
2. For the node level analysis (results in figure 5), any multiple comparison correction was applied? How many nodes in total for the whole brain analysis?
3. What is the projected atlas used in figure 5?
4. Is there any insulin resistance information of the GDM group?
5. Is there any difference between the two groups for the gestational weight gain? If so, it may show the evidence in the discussion of “a reduced ability to discriminate food content”.
6. The authors may accidentally mention Supplementary Table 4 in the manuscript, and the contents are already in ST3.

Reviewer #2

(Remarks to the Author)
This paper explore brain responses to different types of food during pregnancy in women with and without gestational diabetes. They recorded visual evoked potentials in pregnant people at 24-36 weeks of gestation. Brain responses were correlated to HbA1c levels in GDM group.

Overall, the manuscript present a novel exploration in the context of pregnancy and GDM. The methods are described in details, and despite a relative low sample size the authors have controlled as much confounder variables as possible.
With that on mind I have the following general comments that could be addressed in the methods, results and/or discussion

sections:
1. How relevant is the familiarity of the participants with the food presented in the pictures during the experiment? Could it affect the results?
2. Please add participants gestational age and gestational weight gain to table 1.
3. The authors suggest in lines 409-411 that reduced ability to discriminate food may lead to excessive GWG. Could you comment more about GWG in the studied population?
4. Despite HbA1c is a good surrogate marker for average blood glucose levels, could you comment why glycemia was not measured before the experiment?

Some other specific comments:
Introduction:
5. Line 53: orbitofrontal cortex is repeated
6. Line 64: Please define OW

Reviewer #3

(Remarks to the Author)
In this study by et al the authors investigated differences in responses to food pictures varying in fat and carbohydrate content in women with GDM or healthy controls in the third trimester of pregnancy. Visual responses to food pictures have been reported previously to be lower in non- pregnant individuals with obesity but whether pregnancy alters this response was previously unknown. The authors report that women of a health weight she stronger visual evoked potentials to high vs low fat foods, whereas women with GDM do not show this distinction. Neither group shows a difference in VEP to high vs low carbohydrate foods. The study identifies the brain regions activated when presented with a fat or carbohydrate food cue and how this differs between women with GDM and controls. finally, the show that brain responses to food cues correlate to HbA1c levels (but not pre- pregnancy BMI) in women with GDM and not controls. The paper is clear and sets the current study well within the context of previously published work. The figures and tables are clear.

I have the following questions for the authors:
- There is brief mention in the discussion that the reduced VEPs to food cues could be due to insulin levels (presumably that high insulin= insulin resistance which blunts neural processing). Is there information on circulating insulin levels on the women in the study?
- It is mentioned that brain food cues to not correlate to pre- pregnancy BMI but do they correlate to current weight in pregnancy?
- The introduction and discussion mention previous findings in non- pregnant individuals in obesity. What is known about food cue evoked brain responses in non- pregnant individuals with T2DM? That seems more relevant to the current situation given that the GFP correlates to HbA1c but not BMI.
- How do the authors explain the fact that the correlation of GFP to HbA1c is seen in women with GDM only, but GDM women have decreased GFP to food cues (both low and high fat) compared to health weight controls, despite there being a close to significant higher HbA1c levels in the GDM group?

Reviewer #4

(Remarks to the Author)
The article reports, for the first time, an investigation of brain responses to viewing food stimuli during pregnancy, comparing individuals with GDM to healthy controls. The findings showed distinct responses to palatable food images: GDM participants showed less activation, and source estimations localized these effects to brain regions associated with control, attention, and reward. In addition, only in the GDM group, these neural responses were associated with HbA1c levels.
The manuscript is clearly written, offers detailed methodology, along with compelling and meaningful results on brain function during pregnancy. I was personally pleased to see the first results emerging from the previously published protocol, and I found the discussion beginning at Line 431 especially insightful. My comments below mainly consist of questions and suggestions for clarification, rather than criticism.

Comment 1, Introduction
In the first part of the introduction, the authors focus on obesity-related neural mechanisms, then transition to metabolic factors such as type 2 diabetes, and finally introduce pregnancy and GDM. To improve clarity and maintain a clear narrative focus on the pregnant population, it may be helpful to shorten or relocate the section on obesity-related brain characteristics to the Discussion. This would help emphasize from the outset that the manuscript centers on brain function during pregnancy.

Comment 2, Methodology
My understanding is that participants with GDM who were already using glucose lowering treatment were excluded, and that all EEG recordings for the GDM group were conducted before any treatment initiation. If this is correct, Table 1 as currently presented may be confusing. I recommend rephrasing the relevant information for clarity or considering removing the last observation (“Glucose lowering treatment”) from the table.

Comment 3, Methodology
The EEG recordings were conducted 2 hours after a meal. Because women with GDM typically show elevated 2 hour

postprandial glucose levels compared with healthy controls (indeed, this difference is part of the diagnostic criteria) it would be helpful to clarify why this particular time point was chosen. Could you explain the rationale for selecting this window rather than a time when the two groups might have more comparable glucose levels, such as before a meal or after a longer fasting period? Additionally, given the later discussion on hyperglycemia and brain responses, it would be important to address whether this physiological difference between the groups was taken into account when interpreting the findings.

Comment 4, Results
Regarding Table 1, it would be helpful to know how many participants in the GDM group met criteria for obesity, as well as the obesity prevalence in both groups. Including information on gestational weight gain would also provide valuable context for interpreting the metabolic profile of the GDM group.

Comment 5, Results
Did educational level influence the observed brain responses? Based on Table 1, the GDM group appears to have a lower proportion of participants with a university-level education. It would be useful to clarify whether educational level was examined as a potential covariate or whether it had any measurable impact on the neural outcomes.

Comment 6, Discussion
In the discussion section beginning at Line 413, the authors summarize their findings on neural responses and HbA1c and then transition to evidence from studies of food cue reactivity during hyperglycemia in the non-pregnant obese population. This juxtaposition may give the impression of a comparable mechanism, but does not fully articulate how the present findings relate to reported neural characteristics in obesity. Strengthening this connection, or clarifying distinctions, would improve interpretability.

At Line 415, please explicitly indicate that the described association applies specifically to the GDM group. Similarly, in Lines 471–473, please clarify that these statements refer to the GDM population.

Based on the current results, the study supports the interpretation that in individuals with GDM, higher average glucose level (as reflected by HbA1c levels within a near normal range) is associated with stronger neural responses to visual food cues. However, given that EEG recordings were conducted at 2 hours after a meal, when glucose levels are likely to differ systematically between groups, this physiological state may have influenced the observed neural responses. Although HbA1c is a well-validated measure of long-term glycemic control, it may not adequately represent the glycemic status relevant for brain function during the specific testing session, and therefore may not be directly comparable to the previous evidence the authors cite.
For these reasons, I recommend clearly discussing how the present findings align with, or differ from, the existing evidence referenced in the manuscript.

In addition, are there prior data on neural responses to food cue stimulation during hyperglycemia in patients with Type 2 diabetes? Something related was mentioned in the introduction. Integrating such evidence in the Discussion would strengthen the argument, especially because the current study compares GDM with healthy controls rather than obese versus normal-weight participants.

Small comments:
Line 53, OFC is mentioned twice.
Line 64, OW appears for the first time. If it is overweight, please specify.

Version 1:

Reviewer comments:

Reviewer #1

(Remarks to the Author)
My previous comments have been addressed.

Reviewer #2

(Remarks to the Author)
The authors have provided comprehensive responses to all the comments and manuscript looks good for being published.

Reviewer #3

(Remarks to the Author)
The authors have answered my questions in a satisfactory manner. There answers are very thorough and in some cases

contain information that would benefit the reader if included in the discussion, particularly the enhanced discussion on insulin resistance and consequences for the brain.

Reviewer #4

(Remarks to the Author)
I would like to thank the authors for their thoughtful responses to my questions and for incorporating my suggestions. I only have one minor comment in the Introduction section.

Introduction
The Introduction section is very clear and easy to follow. However, lines 120–129 (in the combined PDF) appear to contain elements of the results and conclusions. Please discuss with the editor whether this content is more appropriate for another section.

Methodology
I thank the authors for addressing my question regarding the rationale for selecting the 2-hour postprandial timing. I now understand that potential glucose level differences were considered part of the expected physiological state and were therefore not adjusted for, in order to better reflect real-world metabolic conditions. With the additional explanation provided in the discussion, this point is now clear.
I agree that including acute glycemic measures and pre- and post-meal status would be of interest. However, as noted by the authors, continuous glucose monitoring systems are currently validated primarily for patients with gestational diabetes mellitus (also the case in my country), which makes their application in healthy pregnant populations challenging. I look forward to future studies addressing this aspect.

Results
Page 10, line 358: I appreciate the inclusion of an additional analysis accounting for relevant baseline characteristic differences.

Discussion
The Discussion section has been improved and is now clearer.

Open Access This Peer Review File is licensed under a Creative Commons Attribution 4.0 International License, which permits use, sharing, adaptation, distribution and reproduction in any medium or format, as long as you give appropriate credit to the original author(s) and the source, provide a link to the Creative Commons license, and indicate if changes were made.
In cases where reviewers are anonymous, credit should be given to 'Anonymous Referee' and the source.
The images or other third party material in this Peer Review File are included in the article’s Creative Commons license, unless indicated otherwise in a credit line to the material. If material is not included in the article’s Creative Commons license and your intended use is not permitted by statutory regulation or exceeds the permitted use, you will need to obtain permission directly from the copyright holder.
To view a copy of this license, visit https://creativecommons.org/licenses/by/4.0/

Reviewers' comments:

Reviewer #1 (Remarks to the Author):

This study aimed to investigate the impact of gestational diabetes mellitus (GDM) on maternal brain responses to visual food cues in pregnancy. The association of hbA1c/BMI and VEPs was examined. The authors try to fill in the gap of understanding these conditions effect on the brain responses to visual food cues during pregnancy. The manuscript is easy to follow and well structured.

Major comments:

1. According to Table 1, both groups were well maintained for the hbA1c level, thus yields no significant difference. In the glucose lowering treatment, about 40% were not treated but still well maintained or were they diagnosed using other parameters? So hypothetically, how much effect of GDM is expected to detect with the well maintained hba1c level?

First, we thank the reviewer in general for the thorough and helpful review. Then, we thank the reviewer specifically for this important clarification request. In Switzerland, GDM is diagnosed between 24–28 weeks of gestation using a 75 g oral glucose tolerance test (OGTT) according to the International Association of Diabetes Pregnancy Study Group (2010). Importantly, HbA1c can be a crude indicator of overall glucose control in pregnancy (especially for research; in clinical practice it is rather used for women with pre-existing diabetes who have higher values), but is not used as a diagnostic criterion for GDM. This is also due to hemodilution and a higher turnover the red blood cells, and thus most patients with GDM remain within the normal or slightly elevated range for HbA1c. Participants in the GDM group were recruited and tested very shortly after diagnosis and prior to initiation of any lifestyle and potential glucose-lowering treatment. All participants received, as part of the usual care, lifestyle and potential pharmacological treatment (40%, as mentioned), but only after study participation in pregnancy and all treatment was stopped at delivery.

To avoid confusion regarding "Glucose lowering treatment" previously presented in Table 1, and in line with the suggestion from the other reviewer, we have removed this information, as no participant had initiated pharmacological treatment before the study which includes also the EEG recording (and any subsequent treatment occurred at least two weeks after study participation). Data on the need of glucose lowering treatment was only collected in the postpartum.

2. In figure 4, it is clearer that only 1 participant is in the abnormal range of the hba1c. The entire association of the GDM group is suspected to be driven by that subject (6.5%) and the one about at the upper edge of normal range (slightly higher than 5.5%). With very few subjects have higher value of hba1c, this association may be quite weak. Together with both groups included in the hba1c association analysis, is it still significant?

We thank the reviewer for this observation regarding the distribution of HbA1c values. Figure 4 shows that one participant in the GDM group has an HbA1c value of 6.5%, while a second participant has a value within the normal range (5.6%), which remains within the normal range (up to 5.7% or 6% for out of pregnancy, while there are no clear-defined normal ranges for pregnancy). To address the concern that a single participant may be driving observed associations, we performed a sensitivity analysis excluding the participant with the high HbA1c value (6.5%). The association between HbA1c and brain responses remained significant (all, $p<0.03$), suggesting that the observed relationship is not solely driven by this participant. In pregnancy, there are no defined clinical reference normal ranges for HbA1c, only target values that range between < 6%, 6.5% or even 7%, but this only for individuals with pre-existing diabetes and this depends on the scientific society and the pregnancy stage (American Diabetes Association Professional Practice Committee, 2026 and National Institute for Health and Care Excellence, 2017). Outside of pregnancy, the HbA1c thresholds for prediabetes, the stage above normal and before diabetes, are either ≥5.7% according to the American Diabetes Association and ≥6% according to National Institute of Health and Care Excellence, while the HbA1c-based diagnosis for diabetes is ≥6.5% for both guidelines.

Regarding your question related to the combined analysis: when both the GDM and control groups are pooled, the association is not significant. This suggests the association may be specific and driven by either higher-normal values and not be present in lower-normal values, or it may be potentially influenced by the more insulin-resistant state of participants with GDM. This effect may also be diluted in the combined analysis due to the rather small sample size.

We acknowledge the reviewer's concern regarding the limited number of participants and agree that these findings should be considered exploratory. We have revised the Results (page 10) and Discussion section (page 12) accordingly.

3. Similar problem may be applied to obesity status. The authors also introduced many evidences supporting the impact of obesity in neural processing of food cues, but the participants included were merely overweight, please indicate how many were obese. Or the authors may try to give examples and explain that the subtle overweight can also have an impact. However, such effect was not detected in the current study, and this need to be explored.

We thank the reviewer for this important comment. The pre-pregnancy BMI in the GDM group ranged from 17.2 to 37.7 kg/m^2, with a mean of 27.7 kg/m^2. Regarding the specific distribution, our sample of participants in the GDM group included 1 participant in the underweight range, 5 in the normal weight range, 5 in the overweight range, and 7 participants met the clinical criteria for obesity (BMI ≥30 kg/m^2).

We acknowledge that previous studies have demonstrated effects of both overweight and obesity on brain responses to food cues. We agree that the impact of body weight per see across BMI categories should be further explored in larger samples with stratified analyses. In the present study, pre-pregnancy BMI was included as a covariate in the ANCOVA model assessing brain responses and HbA1c (see page 10) to control for the potential influence of pre-pregnancy BMI. Importantly, the characterizing feature of GDM is primarily defined by their increased glucose values and insulin resistance, which can also be observed in women with GDM with normal BMI (Buchanan & Xiang, 2005). Thus, evidence of obesity in other studies may also be a marker of higher insulin resistance (present also in normal-weight individuals with GDM) and thus most likely brain insulin resistance.

4. Were any covariates controlled in the statistical analyses? Given the evidences of impact of obesity in neural processing of food cues as the authors listed, and the participants have BMI information, was it considered as a confounding factor? Additionally, in table 1, the educational level was significant different between the two groups, was it controlled?

We thank the reviewer for this important comment. To ensure that the observed associations between HbA1c levels and brain responses to food cues were independent of relevant demographic and clinical variables, we performed additional adjusted analyses for variables that differed between groups. Specifically, pre-pregnancy BMI, educational level, and gestational age were included as covariates in the analyses. These adjustments are now reported in the manuscript (see page 10).

Minor comments:

1. Please add the effect size of the significant correlations reported in the abstract.

We thank the reviewer for this suggestion. The effect sizes for the significant correlations have now been added to the abstract.

2. For the node level analysis (results in figure 5), any multiple comparison correction was applied? How many nodes in total for the whole brain analysis?

We thank the reviewer for these remarks and we realize that additional methodological details were needed. Our original analyses included a spatial extent criterion (i.e. minimally 10 contiguous nodes that each met the 0.05 significance criterion). This criterion was based on randomization thresholds determined with Alphasim software (Forman et al., 1995; see also Toepel et al., 2009; De Lucia et al., 2010; Knebel and Murray, 2012; Matusz et al., 2015; Matusz et al., 2016; Retsa et al., 2018 for similar implementations in source modelling analyses). Additionally, we conducted post hoc comparisons with

Bonferroni correction for multiple comparisons to assess within- and between-group differences. The matrix of solution points included a total of 3,058 nodes within the gray matter (see page 9).

3. What is the projected atlas used in figure 5?

We regret any confusion. There is no projected atlas used in Figure 5. Instead, the figure displays results overlaid on the Montreal Neurological Institute (MNI) average brain that was used for generation of the lead field matrix.

4. Is there any insulin resistance information of the GDM group?

We thank the reviewer for this important question. Insulin resistance (IR) measures were not specifically collected for this study. However, it is well established that women with GDM exhibit greater IR compared to healthy counterparts (Buchanan & Xiang, 2005). Given the clinical and pregnancy context, participants were already subject to a high number of medical appointments and assessments. To minimize participant burden, additional procedures were not included, particularly as the study visit had a duration of approximately 3.5 hours. Standard surrogate measures of IR, such as HOMA-IR, would have required a separate fasting visit, which was not feasible within the study design and where recruitment turned out to be difficult. More precise, complex, but also cumbersome measures of IR (such as Matsuda or the insulin-sensitivity-index for example which both last over several hours) would have been needed. This limitation has now been acknowledged in the Discussion section (see pages 12 and 13).

5. Is there any difference between the two groups for the gestational weight gain? If so, it may show the evidence in the discussion of “a reduced ability to discriminate food content”.

We thank the reviewer for this observation. Gestational weight gain (GWG) has now been included as part of the manuscript. No significant differences in GWG were observed between groups. However, given that a substantial proportion of women with GDM were classified as overweight (27%) or obese (39%) based on pre-pregnancy BMI, similar absolute GWG between groups may nonetheless reflect a relatively higher than recommended weight gain in the GDM group prior to the study visit. This has been addressed in the Discussion (see pages 11 and 12).

6. The authors may accidentally mention Supplementary Table 4 in the manuscript, and the contents are already in ST3.

We thank the reviewer for this observation. This has now been corrected, and the reference to Supplementary Table 4 has been modified to Supplementary Table3.

Reviewer #2 (Remarks to the Author):
This paper explore brain responses to different types of food during pregnancy in women with and without gestational diabetes. They recorded visual evoked potentials in pregnant people at 24-36 weeks of gestation. Brain responses were correlated to HbA1c levels in GDM group.

Overall, the manuscript present a novel exploration in the context of pregnancy and GDM. The methods are described in details, and despite a relative low sample size the authors have controlled as much confounder variables as possible.
With that on mind I have the following general comments that could be addressed in the methods, results and/or discussion sections:
1. How relevant is the familiarity of the participants with the food presented in the pictures during the experiment? Could it affect the results?

First, we thank the reviewer in general for the thorough and helpful review. We specifically thank the reviewer for raising this important point. Prior research has indeed shown that exposure and learned associations can modulate neural responses to food cues (e.g., Small et al., 2004), suggesting that familiarity may influence food cue processing. In the present study, the stimuli consisted of commonly consumed foods. While familiarity was not explicitly assessed, the pictures used in this experiment have previously been rated as highly familiar (Blechert et al., 2019), and included the use of commonly familiar food pictures in non-vegetarians (e.g., strawberries, cake, burger), which reduces the probability that systematic differences in familiarity substantially influenced the observed effects. Nevertheless, we acknowledge that individual differences in familiarity and preference could modulate brain responses, and would be recommended for future studies to assess these factors.

2. Please add participants gestational age and gestational weight gain to table 1.

We thank the reviewer for this suggestion. Gestational age and gestational weight gain have now been included in Table 1.

3. The authors suggest in lines 409-411 that reduced ability to discriminate food may lead to excessive GWG. Could you comment more about GWG in the studied population?

We thank the reviewer for this comment. Information on gestational weight gain has now been included in the manuscript, and no differences in GWG were observed between groups. Further discussion on GWG has been added on page 11. Importantly, we measured the ability to discriminate food in late pregnancy, when a substantial proportion of GWG has already occurred. Evidence outside of pregnancy suggests that altered brain responses to food cues are associated with body weight regulation and eating behavior (Chechlacz et al., 2009; Batterink et al., 2010; Murdaugh et al., 2012; Yokum & Stice, 2019). However their relevance to GWG during pregnancy remains unclear and requires longitudinal studies and

larger sample sizes to determine whether brain responses are associated with subsequent GWG.

4. Despite HbA1c is a good surrogate marker for average blood glucose levels, could you comment why glycemia was not measured before the experiment?

We thank the reviewer for this comment. Glycemia was not measured in this study in order to minimize participant burden. Given the clinical context of pregnancy, particularly relevant for women with GDM, participants were already undergoing a high number of medical appointments and assessments during pregnancy. The study visit itself lasted approximately 3.5 hours, and additional metabolic measurements (e.g., fasting glucose) would have increased the procedural and logistical burden.

We acknowledge that more thorough glycemic measures, such continuous glucose monitoring, could provide complementary information. However, current CGM monitoring systems available in Switzerland are only validated in patients with diabetes (and thus higher glucose levels), and their accuracy may be reduced in normo or near-normoglycemic ranges, particularly during pregnancy where physiological changes such as water retention may further influence subcutaneous sensor readings. In addition, we have shown the initial days of sensor may provide less accurate measurements, probably related to local inflammation around the sensor site (Treviño Montemayor et al., 2026). Fasting glucose was also not assessed, as it may be sensitive to short-term dietary and overnight variability, and would require repeated measurements to obtain a more robust result and would be also less discriminative than post-challenge glucose levels which in turn are again more difficult to obtain in a standardized way within the study protocol (would require a standardized weight adapted meal and exact and identical postprandial timing).

Some other specific comments:

Introduction:

5. Line 53: orbitofrontal cortex is repeated

We thank the reviewer for this observation, the repetition has been corrected in the manuscript.

6. Line 64: Please define OW

We thank the reviewer for this comment. OW has now been introduced in the manuscript (see page 4).

Reviewer #3 (Remarks to the Author):
In this study by et al the authors investigated differences in responses to food pictures varying in fat and carbohydrate content in women with GDM or healthy controls in the third trimester of pregnancy. Visual responses to food pictures have been reported previously to be lower in non- pregnant individuals with obesity but whether pregnancy alters this response was previously unknown. The authors report that women of a health weight she stronger visual evoked potentials to high vs low fat foods, whereas women with GDM do not show this distinction. Neither group shows a difference in VEP to high vs low carbohydrate foods. The study identifies the brain regions activated when presented with a fat or carbohydrate food cue and how this differs between women with GDM and controls. finally, the show that brain responses to food cues correlate to HbA1c levels (but not pre- pregnancy BMI) in women with GDM and not controls. The paper is clear and sets the current study well within the context of previously published work. The figures and tables are clear.

I have the following questions for the authors:
- There is brief mention in the discussion that the reduced VEPs to food cues could be due to insulin levels (presumably that high insulin= insulin resistance which blunts neural processing). Is there information on circulating insulin levels on the women in the study?

> *First, we would like to thank the reviewer in general for the thorough and helpful review. Insulin resistance measures were not collected in this study as, in the clinical context of pregnancy, particularly relevant for women with GDM, participants were already undergoing a high number of medical appointments and assessments during pregnancy and many had symptoms of late pregnancy (tiredness etc.) and thus recruitment was challenging. Given the duration of the study visit (approximately 3.5 hours), additional metabolic assessments (e.g., fasting glucose) would have needed more study visits and increased the procedural and logistical demands. We acknowledge that future studies should consider including measures of insulin concentration and sensitivity. Standard surrogate measures of IR, such as HOMA-IR, would have required a separate fasting visit, which was not feasible within the study design. More precise, complex, but also cumbersome measures of IR (such as Matsuda or the insulin-sensitivity-index for example which both last over several hours) would have been needed. However, it is well established that women with GDM exhibit higher IR compared to healthy controls, independent of their BMI (Buchanan & Xiang, 2005). This supports the interpretation that differences in brain responses observed in the present study may be related to underlying metabolic differences associated with GDM (see page 12).*

- It is mentioned that brain food cues to not correlate to pre- pregnancy BMI but do they correlate to current weight in pregnancy?

We thank the reviewer for this relevant question. We performed additional analyses to investigate the association between current weight during pregnancy and brain responses to food cues. No significant interaction was observed.

- The introduction and discussion mention previous findings in non- pregnant individuals in obesity. What is known about food cue evoked brain responses in non- pregnant individuals with T2DM? That seems more relevant to the current situation given that the GFP correlates to HbA1c but not BMI.

We thank the reviewer for this insightful comment. In response, we have included evidence from studies in non-pregnant individuals with T2DM in the Introduction and Discussion. These studies show increased responses to food cues in regions such as the insula, orbitofrontal cortex, and basal ganglia, which are involved in reward processing and salience compared to healthy controls. One has to be careful as glycemic levels are substantially higher in individuals with T2DM than those with GDM. In addition, both obesity and GDM are related to insulin resistance which in turn is related to brain insulin resistance which impacts of brain responses to food cues (Heni et al., 2015). We mention this now in the MS, see page 12.

- How do the authors explain the fact that the correlation of GFP to HbA1c is seen in women with GDM only, but GDM women have decreased GFP to food cues (both low and high fat) compared to health weight controls, despite there being a close to significant higher HbA1c levels in the GDM group?

We thank the reviewer for this question. Importantly, no main effect of group was observed in TW1., indicating that overall brain responses to food cues were not significantly lower in women with GDM compared healthy controls. However, we observed a significant interaction between fat x carb x group x hbA1c, driven by an interaction fat x carb x hbA1c within the GDM group only. A possible explanation for these results is that brain responses to food cues are modulated by several different, both acute and chronic metabolic factors and are subject to both glucose measures as well as insulin and insulin resistance (and both chronic glucose measure and insulin resistance are related to subacute and chronic food intake which also modulates brain responses). Thus, evidence from previous studies outside of pregnancy suggest that brain responses to food cues are strongly modulated by both acute and chronic metabolic state, including glucose and insulin dynamics (e.g., Martens et al., 2013; Heni et al., 2014), and insulin plays a central role in regulating food related brain activity (Heni et al., 2015). In insulin-resistant states, this central effect is attenuated, leading to altered coupling between peripheral metabolic status and brain responses, as also shown in obesity and diabetes-related neuroimaging studies (Anthony et al., 2006; Belfort-DeAguiar et al., 2018). Women with GDM generally have a higher

IR compared to healthy controls and this is also seen in normal-weight women with GDM (Buchanan & Xiang, 2005).Within this study, HbA1c may capture more chronic inter-individual differences in long-term glycemic exposure and metabolic dysregulation within the GDM group, which relates to brain responsiveness to food cues. Our data show that in HC, with usually normal insulin resistance, lower HbA1c is associated with tendency to higher GFP and this relationship is probably dysregulated in individuals with GDM (reflecting a different brain process that may be related to the present insulin resistance).

In analogy, in acute situations, mild hyperglycemia of around 7.2 mmol/l (as frequently observed in the postprandial state) is related to lower activity in the hypothalamus and putamen in individuals with NW compared to normoglycemia, while those with OB exhibited increased activation in reward-related regions, such as the insula, putamen, and prefrontal cortex, without any suppressive effect on hypothalamic activity (Belfort-DeAguiar et al., 2018).

Reviewer #4 (Remarks to the Author):
The article reports, for the first time, an investigation of brain responses to viewing food stimuli during pregnancy, comparing individuals with GDM to healthy controls. The findings showed distinct responses to palatable food images: GDM participants showed less activation, and source estimations localized these effects to brain regions associated with control, attention, and reward. In addition, only in the GDM group, these neural responses were associated with HbA1c levels.
The manuscript is clearly written, offers detailed methodology, along with compelling and meaningful results on brain function during pregnancy. I was personally pleased to see the first results emerging from the previously published protocol, and I found the discussion beginning at Line 431 especially insightful. My comments below mainly consist of questions and suggestions for clarification, rather than criticism.

Comment 1, Introduction
In the first part of the introduction, the authors focus on obesity-related neural mechanisms, then transition to metabolic factors such as type 2 diabetes, and finally introduce pregnancy and GDM. To improve clarity and maintain a clear narrative focus on the pregnant population, it may be helpful to shorten or relocate the section on obesity-related brain characteristics to the Discussion. This would help emphasize from the outset that the manuscript centers on brain function during pregnancy.

> *We thank the reviewer for the very encouraging words, the overall comments and specifically for this suggestion. The Introduction has been revised to improve clarity and maintain a stronger focus on pregnancy. Specifically, the section on obesity-related neural mechanisms has been shortened and reframed to better support the study pregnancy context.*

Comment 2, Methodology
My understanding is that participants with GDM who were already using glucose lowering treatment were excluded, and that all EEG recordings for the GDM group were conducted before any treatment initiation. If this is correct, Table 1 as currently presented may be confusing. I recommend rephrasing the relevant information for clarity or considering removing the last observation ("Glucose lowering treatment") from the table.

> *We thank the reviewer for this helpful observation. We agree that all participants with GDM were studies before any lifestyle or medical treatment initiations and that therefore the presentation of this information in Table 1 could be confusing. To improve clarity and avoid reporting information not directly relevant to the analyses, the "glucose-lowering treatment" variable has now been removed.*

Comment 3, Methodology
The EEG recordings were conducted 2 hours after a meal. Because women with

GDM typically show elevated 2 hour postprandial glucose levels compared with healthy controls (indeed, this difference is part of the diagnostic criteria) it would be helpful to clarify why this particular time point was chosen. Could you explain the rationale for selecting this window rather than a time when the two groups might have more comparable glucose levels, such as before a meal or after a longer fasting period? Additionally, given the later discussion on hyperglycemia and brain responses, it would be important to address whether this physiological difference between the groups was taken into account when interpreting the findings.

> *We thank the reviewer for this important question. The 2-hour post-meal time point was selected to ensure a standardized postprandial and satiated metabolic state across participants, reducing additional factors related to current hunger. In addition, during pregnancy, prolonged fasting may lead to nausea, even in later pregnancy. When fasting over a more prolonged period many women also feel dizzy and uncomfortable. Shorter fasting period, e.g. skipping lunch, can be difficult to interpret due to pregnancy-related gastroparesis and to implement due to the fact that many pregnant women do not feel comfortable to skip a meal. This is relevant as the study visit itself lasted approximately 3.5 hours. As the reviewer mentions, the condition was also selected because it represents a physiologically and clinically relevant state during which metabolic differences between women with GDM and controls would be highlighted, particularly related to the glucose levels. We acknowledge that this probably introduces more accentuated differences in glycemic levels at the time of testing (and thus observed discrepancies are linked to both chronic and acute differences), this reflects a real-world metabolic state in which brain responses to food cues occur during a large proportion of the day. In a future study, we aim, if feasible, to assess both pre- and post-ingestion states to provide a more comprehensive characterization of state-dependent metabolic influences on the brain responses during pregnancy. We also acknowledge as a limitation the absence of concurrent acute glycemic measures. We acknowledge that more thorough glycemic measures including ideally continuous glucose monitoring (with the downside that the reliability is much less reliable in near-normo/normo glycemic levels and existing sensors (in Switzerland) are only validated for patients with diabete)s. For better validity, the glucose monitoring device should be placed 2-3 days before study start as initial local inflammation around the sensor site may reduce the accuracy substantially (Treviño Montemayor et al., 2026). This challenges feasibility issues even more.*

Comment 4, Results

Regarding Table 1, it would be helpful to know how many participants in the GDM group met criteria for obesity, as well as the obesity prevalence in both groups. Including information on gestational weight gain would also provide valuable context for interpreting the metabolic profile of the GDM group.

We thank the reviewer for this suggestion. The distribution of participants across BMI categories (n, %) in the GDM group has now been reported in the Results section (see page 9). In addition, information on gestational weight gain up to the time of the study visit has been included in Table 1. Additional discussion regarding GWG has been included in the Discussion section (see page 11 & 12).

Comment 5, Results
Did educational level influence the observed brain responses? Based on Table 1, the GDM group appears to have a lower proportion of participants with a university-level education. It would be useful to clarify whether educational level was examined as a potential covariate or whether it had any measurable impact on the neural outcomes. To check

We thank the reviewer for this important comment. To address the specific question regarding educational level and to ensure that the observed associations between HbA1c levels and brain responses to food cues were independent of potential confounding factors, we have now adjusted our analyses for relevant variables that differed between groups. Specifically, pre-pregnancy BMI, educational level, and gestational age were included as covariates in the analyses. These adjustments are now reported in the manuscript (see page 10).

Comment 6, Discussion
In the discussion section beginning at Line 413, the authors summarize their findings on neural responses and HbA1c and then transition to evidence from studies of food cue reactivity during hyperglycemia in the non-pregnant obese population. This juxtaposition may give the impression of a comparable mechanism, but does not fully articulate how the present findings relate to reported neural characteristics in obesity. Strengthening this connection, or clarifying distinctions, would improve interpretability.

We thank the reviewer for this important comment. We have revised the Discussion to better clarify the relationship between our findings and previous. In particular, we now more explicitly distinguish between the evidence from non-pregnant populations, including studies with individuals with T2DM and such with obesity, and the present findings in pregnant women with and without GDM. We have clarified that while prior studies in both obesity and in hyperglycemia describe changes in brain responses to food cues in reward- and homeostatic-related regions, these findings are observed in a different and should not be interpreted as reflecting the same underlying mechanism in pregnancy. Instead, we highlight that these studies provide a relevant metabolic framework for interpreting how metabolic state, glycemic levels and variation may be associated with brain responses to food cues, while our results extend this evidence to pregnancy (see page 11).

At Line 415, please explicitly indicate that the described association applies specifically to the GDM group. Similarly, in Lines 471–473, please clarify that these statements refer to the GDM population.

We thank the reviewer for this suggestion. The manuscript has been revised to explicitly indicate that the reported association at Line 415 pertains specifically to the GDM group. Similarly, the relevant statements in Lines 471–473 have been clarified to specify that they refer to the GDM population.

Based on the current results, the study supports the interpretation that in individuals with GDM, higher average glucose level (as reflected by HbA1c levels within a near normal range) is associated with stronger neural responses to visual food cues. However, given that EEG recordings were conducted at 2 hours after a meal, when glucose levels are likely to differ systematically between groups, this physiological state may have influenced the observed neural responses. Although HbA1c is a well-validated measure of long-term glycemic control, it may not adequately represent the glycemic status relevant for brain function during the specific testing session, and therefore may not be directly comparable to the previous evidence the authors cite.
For these reasons, I recommend clearly discussing how the present findings align with, or differ from, the existing evidence referenced in the manuscript.

We thank the reviewer for this important and insightful comment. The 2-hour post-meal time point was chosen to ensure a standardized postprandial and satiated metabolic state across participants, thereby reducing factors related to hunger due to fasting status. We agree that the postprandial state may have potentially contributed to and accentuated the observed brain responses, and we have now clarified this in the Discussion (see page 11). Importantly, HbA1c reflects average glycemic exposure over the preceding 2–3 months. Women were instructed to eat a balanced meal 2h before and this was checked with them. In these high normal HbA1c values, the postprandial glucose values (as opposed to fasting values) contribute to the large majority of HbA1c value (that contribution increases with decreasing HbA1c and is already above 70% in HbA1c values around 7%) therefore HbA1c remains relevant for postprandial glucose values in general and the interpretation of the observed associations (Monnier & Colette., 2010). In future studies, we aim to assess both pre- and post-ingestion states to more comprehensively characterize state-dependent metabolic influences on brain responses. We have now clarified in the Discussion our findings and how these align or differ from the existing evidence (see page 12).

In addition, are there prior data on neural responses to food cue stimulation during hyperglycemia in patients with Type 2 diabetes? Something related was mentioned in the introduction. Integrating such evidence in the Discussion would strengthen the argument, especially because the current study compares GDM with healthy controls rather than obese versus normal-weight participants.

We thank the reviewer for this insightful comment. In response, we have expanded the Discussion to include evidence from studies in non-pregnant individuals with T2DM, although glycemic values in this population are substantially higher than those observed in individuals with GDM. These studies show increased responses to food cues in regions such as the insula, orbitofrontal cortex, and basal ganglia, which are involved in reward processing and salience compared to healthy controls (see page11).

Small comments:
Line 53, OFC is mentioned twice.

We thank the reviewer for pointing this out. This has now been corrected.

Line 64, OW appears for the first time. If it is overweight, please specify.

We thank the reviewer for this remark. The abbreviation has now been defined in the manuscript.
