# Supplementary material for: Brain responses to food cue viewing in pregnant women with and without gestational diabetes mellitus
Source: Commun Med (Lond). 2026 Aug 6;6:426. doi: 10.1038/s43856-026-01792-3 (PMC13448487; doi:10.1038/s43856-026-01792-3)
Supplement: Supplementary file 2 [file 43856_2026_1792_MOESM2_ESM.pdf]

Supplementary tables and figures

Supplementary Table 1. Comparison of women with and without valid EEG data.

Variable	Women excluded for analyses (N=32)	Women included for analyses (N=39)	Mean difference	*p-value*
**Age (years)**	33.21 ± 4.61	31.67 ± 3.91	-1.55 ± 4.29	0.128
**Pre-pregnancy weight (kg)**	62.78 ± 12.22	66.46 ± 14.25	3.68 ± 13.41	0.253
**Pre-pregnancy BMI (kg/m2)**	23.28 ± 4.00	24.36 ± 5.38	1.08 ± 4.80	0.348
**Weight (kg)**	73.90 ± 12.85	76.48 ± 13.64	2.58 ± 13.25	0.414
**HbA1c (%)**	5.17 ± 0.35	5.07 ± 0.35	-0.11 ± 0.35	0.204

Supplementary Table 2. Group-averaged reaction time for women with GDM and healthy controls and food/non-food category.

Group	Condition	Median RT (ms)	IQR (ms)	Test	Statistic	*p-value*	η²
**GDM**	Food	482	53.5	Wilcoxon	Z = -3.72	<0.001	0.9
	Non-Food	550.1	87.4				
	HFHC	478	52.3	Friedman	$\chi^2(3) = 7.53$	0.06	-
	HFLC	483.9	54.9				
	LFHC	486.5	48.5				
	LFLC	488.7	54.7				
**HCO**	Food	496.6	78	Wilcoxon	Z = -3.98	<0.001	0.9
	Non-Food	583.5	63.7				
	HFHC	487.1	76.1	Friedman	$\chi^2(3) = 18.60$	<0.001	0.3
	HFLC	501	77.5				
	LFHC	492.8	94.6				
	LFLC	494.8	62.1				
**Between Groups**	Food	-	-	Mann-Whitney U	U = 177	0.74	-
	Non-Food	-	-	Mann-Whitney U	U = 133	0.12	-
	HFHC	-	-	Mann-Whitney U	U =172	0.63	-
	HFLC	-	-	Mann-Whitney U	U = 164	0.48	-
	LFHC	-	-	Mann-Whitney U	U = 177	0.74	-
	LFLC	-	-	Mann-Whitney U	U = 182	0.84	-

Supplementary Table 3. LAURA source estimations over the TW1 and TW2 regarding viewing of food pictures of different carbohydrate and fat content.

Time window	Interaction	MNI* Coordinates	Brain region	*Post hoc* summary[&]
**TW1 127-155 ms**	Fat x Group	44, -68, -12	Right visual association cortex	HCO: HF>LF<br>GDM: NS
		52, -60, 20	Right angular gyrus	HCO: HF>LF<br>GDM:NS
		44, 12, 28	Right inferior frontal gyrus (pars opercularis)	HCO: NS<br>GDM: HF<LF
	Carb x Group	-20, 4, -4	Left globus pallidus	HCO: HC>LC<br>GDM: NS<br>GD[+]: HCO>GDM for HC
		-4, -76, 52	Left visual-motor area	HCO: NS<br>GDM: HC<LC<br>GD[+]: HCO>GDM for HC
		4, 36, 36	Right frontal eye fields	HCO: HC>LC<br>GDM: NS
		20, 68, -4	Right anterior prefrontal cortex	HCO: NS<br>GDM: HC>LC<br>GD[+]: HCO>GDM for LC
	Fat x Carb x Group	28, -92, 4	Right secondary visual cortex	HCO: HFHC<HFLC; HFHC< LFHC; HFLC>LFLC<br>GDM:HFHC>HFLC
		-60, -36, 44	Left supramarginal gyrus	HCO: NS<br>GDM: HFLC< LFLC
		12, 60, 12	Right anterior prefrontal cortex	HCO: HFHC<HFLC<br>GDM:HFHC>HFLC<br>GD[+]: HCO>GDM for HFLC
		-28, 28, 52	Left front eye fields	HCO: HFHC>HFLC<br>GDM: HFHC<LFHC
**TW2 296-361 ms**	Fat x Group	-52, -4, -20	Left middle temporal gyrus	HCO:HF<LF<br>GDM: NS
		-35.92, -4, 60	Left premotor and supplementary motor areas	HCO: HF>LF<br>GDM: NS
	Carb x Group	-36, -68, 52	Left visual-motor area	HCO: HC<LC<br>GDM: NS
		20, 52, 36	Right dorsolateral prefrontal cortex	HCO: HC>LC<br>GDM:HC<LC
	Fat x Carb x Group	36, -84, 12	Right visual association cortex	HCO: NS<br>GDM: HFHC>HFLC
		12, -52, 28	Right ventral posterior cingulate cortex	HCO:HFHC<HFLC<br>GDM: HFHC>HFLC<br>GD[+]: HCO>GDM for HFLC
		28, -4, 4	Right Putamen	HCO: HFHC<HFLC;<br>HFLC>LFLC<br>GDM:NS

* Montreal Neurological Institute [+] Group Difference
[&] Results for *post hoc* comparisons with Bonferroni correction were considered significant at $p<0.05$
NS: not significant
