# Supplementary material for: Brain responses to food cue viewing in pregnant women with and without gestational diabetes mellitus
Source: Commun Med (Lond). 2026 Aug 6;6:426. doi: 10.1038/s43856-026-01792-3 (PMC13448487; doi:10.1038/s43856-026-01792-3)
Supplement: Supplementary file 3 [file 43856_2026_1792_MOESM3_ESM.pdf]

Description of Additional Supplementary Files

File name: Supplementary Data 1

Description: The source data behind the graphs in the paper
